# Physical and mental health characteristics related to trust in and intention to receive COVID-19 vaccination: results from a Korean community-based longitudinal study

**DOI:** 10.4178/epih.e2022064

**Published:** 2022-08-03

**Authors:** Ye Jin Jeon, Youngrong Lee, Ji Su Yang, Young Su Park, Sun Jae Jung

**Affiliations:** 1 Department of Public Health, Graduate School, Yonsei University, Seoul, Korea; 2 Department of Preventive Medicine, Yonsei University College of Medicine, Seoul, Korea; 3 Department of Health Studies, Haverford College, Haverford, PA, USA

**Keywords:** COVID-19, Vaccine, Intention, Public health

## Abstract

**OBJECTIVES:**

The aim of this study was to explore factors affecting attitudes toward coronavirus disease 2019 (COVID-19) vaccination, including socio-demographic characteristics and mental health status during the pandemic.

**METHODS:**

This study analyzed responses from 1,768 participants who were originally included in a community cohort study and responded to 3 online surveys related to COVID-19 (March 2020 to March 2021). The k-means method was used to cluster trust in and intention to receive COVID-19 vaccination. Baseline (2013-2018) socio-demographic characteristics, physical health status, and depressive symptoms were analyzed as exposure variables, and current mental health status was included in the analyses.

**RESULTS:**

Almost half of all participants were classified into the moderate trust and high intention cluster (n=838, 47.4%); those with high trust and high intention accounted only for 16.9%. They tended to be older, had high-income levels, and engaged in regular physical activity at baseline (p<0.05), and their sleep quality and psychological resilience were relatively high compared to other groups.

**CONCLUSIONS:**

Our results suggest that more efforts are required to enhance the perceived need for and trust in COVID-19 vaccination.

## INTRODUCTION

Although various clinical trials (phases 1-3) of coronavirus disease 2019 (COVID-19) vaccines have been actively conducted, and some countries have vaccinated most of their populations with at least 1 dose, the public trust in and intention to receive COVID-19 vaccination remain questionable. Vaccine hesitancy stems from a mistrust of COVID-19 vaccines and, due to social, political, and psychological factors, has evolved into an anti-vaccine movement [1-3]. A global social listening study that considered 5 cities (New York, London, Mumbai, Sao Paulo, and Beijing; June 13 to July 31, 2020) found that COVID-19 vaccine hesitancy was prevalent worldwide (12-38%), and that negative vaccine-related content attracts higher engagement on social media [4]. Vaccine hesitancy, though not limited to COVID-19, has steadily increased, particularly in East Asia and some European countries, where perceptions of the safety and effectiveness of vaccines are lower than in other countries (agreement rate under 50.0%) [5]. In Korea, studies have explored the factors influencing vaccine hesitancy or vaccination intention [6,7].

The COVID-19 pandemic is expected to continue to impose a substantial burden on morbidity and other health-related outcomes (sedentary behavior, depression, exhaustion, and anxiety) and severely disrupt health systems worldwide [8]. Despite the social and health-related effects of COVID-19, and because of low belief in and compliance with vaccination due to concerns about adverse effects, the vaccination rate in Korea in the early stages of vaccine roll-out was relatively low compared to other advanced countries (August 21, 2021; Korea, 22.5%; Japan, 40.2%; Israel, 59.4%; United States, 53.2%; world average, 24.4%).

Although many individual-level characteristics could affect vaccine hesitancy, previous studies have suggested that socio-demographic characteristics impact COVID-19 vaccine hesitancy or refusal [9-12]. According to a study conducted in Italy (January 2021; n= 1,011), younger age, female, low educational level, low income, and absence of comorbidities were significantly associated with vaccine hesitancy [9]. In another United States study, including a community sample of African-Americans (May 28 to June 8, 2020; n = 5,009), the likelihood of vaccine refusal was higher for African-Americans, female, and individuals who had conservative political tendencies [10].

Another characteristic that could affect vaccine refusal is mental health status during the COVID-19 pandemic. Vaccine hesitancy and mental health have mostly been assessed in patients with mental illnesses or disorders, with studies mostly reporting low intention in patient populations and describing management strategies [13], and few studies have been conducted in general populations. Because the willingness to receive COVID-19 vaccination is related to recognition of the collective importance of the decision and trust in COVID-19 prevention, adverse events after vaccination, and stress derived from the COVID-19 pandemic, negative mental health status during pandemic could be related to low hesitancy in the community setting. According to previous studies, various dimensions of psychological factors or mental health-related status could be associated with intention to receive COVID-19 vaccination and trust in the vaccines. A previous study explored psychological characteristics associated with COVID-19 vaccine hesitancy (Ireland, 1,041; United Kingdom, 2,025) and found that individuals who were hesitant toward vaccination had significantly different personalities than others. COVID-19 vaccine hesitancy was found to be related to low agreeableness and a lower tendency to help or care about others in Ireland and the United Kingdom. A United States study also reported that psychological factors explained about 11% of vaccine hesitancy [14]. A previous study conducted in Australia reported that social distancing was significantly related to vaccine hesitancy (n= 7,678) [15]. On the contrary, a previous study reported no significant association between mental health status and vaccine hesitancy (United Kingdom, November to December, 2020; n= 12,035). However, that study only included pre-pandemic anxiety and depressive symptoms [16]. Since many people’s mental health status has changed after the start of the pandemic [17], it is necessary to investigate the association between mental health status during the pandemic and COVID-19 vaccine hesitancy. By identifying attitudes toward vaccination according to mental health in community-based participants, more aspects of vaccine hesitancy or refusal could be understood.

Identifying individuals who refuse or avoid vaccinations due to unclear superstitions and encouraging them to get vaccinated has implications for improving community health [18]. In this study, we aimed to determine whether individual characteristics negatively affect perceptions or refusal of the COVID-19 vaccine through clustering methods. Trust and intention to receive the COVID-19 vaccine are correlated, but there has been no attempt to divide participants into corresponding subgroups using COVID-19 vaccinerelated questions, and this association has not yet been validated. Therefore, we applied the k-means method to cluster participants considering both intention and trust toward the vaccine using a data-driven method involving the Euclidian distance between 2 questions. Furthermore, we explored the relationship between mental health status in the community (after the COVID-19 outbreak) and perceptions of COVID-19 vaccine efficacy.

## MATERIALS AND METHODS

We collected data from a previously conducted prospective cohort, the Cardiovascular and Metabolic Etiology Research Center (CMERC) study, which enrolled urban and suburban (Seoul, Incheon, and Gyeonggi Province) community-dwelling people aged 30-64 years at baseline (baseline enrolment period: 2013-2018; n= 4,060). Details of the cohort have been described elsewhere [19,20]. After the COVID-19 outbreak in Korea (January 20, 2020), researchers conducted 3 consecutive online surveys of eligible CMERC participants (n= 3,913) in March and September 2020 and in March 2021 [21].

The online surveys were conducted to assess the perception of the COVID-19 pandemic and its short-term and long-term impact on mental health status. Questions about trust in and intention to receive COVID-19 vaccination were only included in the third survey (February 19 to March 12, 2021) (Supplementary Material 1). In this analysis, we included the baseline data of the CMERC cohort and the last survey data regarding COVID-19 experiences. Among the survey participants (n= 1,791), 23 were excluded due to missing information regarding the COVID-19 vaccine. Finally, 1,768 participants (mean age at baseline, 50.8 years; male; n= 613, 34.7%) were included in the analyses. The characteristics of individuals who were included in current analyses are shown in Supplementary Material 2 the included individuals tend to be younger, higher-income, and highly educated, and to have better health-related lifestyles, a higher proportion of disease-free status, and better social network status at baseline (p< 0.05).

### The assessment of trust in and intention to receive COVID-19 vaccination

Both trust in and intention to receive COVID-19 vaccination were assessed using a questionnaire developed based on previous research [8,22]. Trust in the vaccine was recorded using the response to the question: “How much do you trust the effects of the COVID-19 vaccine?”, rated on a 6-point Likert scale, ranging from 0 (strongly unreliable) to 5 (strongly reliable). Intention to receive vaccination was reported using a multiple-choice question: “When a vaccine for COVID-19 is approved and widely available to anyone who wants it, would you like to get the vaccine?” Answer choices included: (1) “get the vaccine as soon as you can”; (2) “wait until it has been available for a while to see how it is working for other people”; (3) “only get the vaccine if you are required to do so for work, school, or other activities”; (4) “do not know”; and (5) “refuse to receive the vaccine.” To cluster trust in and intention to receive COVID-19 vaccination, k-means clustering was conducted based on Gower’s distances [23].

### Socio-demographic and clinical information at baseline survey

The baseline assessment (2013-2018) of the CMERC study included demographic characteristics, health behaviors, disease history, psychological conditions, anthropometric measurements, and biochemical analyses of a fasting blood sample.

Socio-demographic factors, such as age at baseline survey, sex, education attainment years ( ≤ 6, 7-9, 10-12, and > 12 years), household income (grouped into quartiles), and marital status (“never married,” “married, living with spouse,” “divorced,” or “widowed.”), were queried.

Cigarette smoking and alcohol consumption were categorized as “never,” “past,” or “current use.” Physical activity was assessed using the Korean version of the International Physical Activity Questionnaire (short form), which enquired about the frequency of each of the following activities: walking, moderate-intensity activity, and vigorous activity (e.g., moderate-intensity activity: carrying light loads, bicycling at a regular pace, or doubles tennis; vigorous activity: heavy lifting, digging, and aerobics). Regular physical activity was defined as at least 150 minutes of moderate or vigorous activity per week [24].

Chronic disease history at baseline was assessed using the standardized question, “Have you ever been diagnosed with any of the listed conditions by a physician?” Comorbidities included the following conditions: stroke, transient ischemic stroke, myocardial infarction, angina, heart failure, chronic renal failure, hypertension, dyslipidemia, diabetes, thyroid disorders, fatty liver disease, chronic hepatitis, liver cirrhosis, asthma, chronic obstructive pulmonary disease, osteoporosis, arthritis, autoimmune disease, and cancer. Diabetes mellitus and hypertension were defined by a diagnosis history (yes), current medication use (yes), or fasting glucose (126 mg/dL) or blood pressure (130/80 mmHg) measures. Body mass index (BMI) was calculated as weight divided by height squared (kg/m2). Upper arm blood pressure was measured 3 times after the participant had been seated and at rest for at least 5 minutes. We used the average of the second and third blood pressure measurements.

Depressive symptoms were assessed using the Korean version of the Beck Depression Inventory-II (BDI-II), a 21-question multiple-choice self-report inventory that is one of the most widely used psychometric tests for measuring the severity of depression. The validity of the Korean version of the BDI-II has been previously verified (area under the curve, 0.93) [25]. Cognitive function was tested using the Korean version of the Mini Mental State Examination for Dementia Screening (MMSE-DS), administered by trained interviewers to participants aged at least 50 years. The validity of the Korean version of the MMSE-DS was verified in a previous study (area under the curve, 0.90) [26].

### Measurements of mental health status during the COVID-19 pandemic

In the online survey, sleep quality, anxiety, post-traumatic stress, depression, loneliness, and psychological resilience scale were included to assess current mental health status during the COVID-19 pandemic. The measurement tool for each mental health domain was as follows: sleep quality: the Korean version of the Pittsburgh Sleep Quality Index (PSQI-K; sensitivity, 0.94; specificity, 0.84) [27]; anxiety: Generalized Anxiety Disorder-7 (GAD-7; area under the curve, 0.91) [28]; post-traumatic stress: Post-traumatic Stress Disorder Checklist for the Diagnostic and Statistical Manual Disorders (fifth edition) (PCL-5; Cronbach’s α= 0.91) [29]; depression: Patient Health Questionnaire-9 (PHQ-9; Cronbach’s α= 0.81) [30]; loneliness: the short version of the UCLA Loneliness Scale (ULS-6) [31]. For all these instruments, a higher score indicates greater severity of each symptom. Psychological resilience was assessed by the short version of the Connor-Davidson Resilience Scale (CD-RISC-10; Cronbach’s α= 0.95) [32,33]; a higher CD-RISC-10 score indicates higher psychological resilience. All mental health indices, except for ULS-6, were verified in the Korean population. For statistical analysis, we used the total score (continuous) as exposure.

### Statistical analysis

K-means clustering, using Gower’s distance and the Forgy algorithm [34], was used to cluster vaccine-related questionnaires for the participants (n= 1,768). According to previous findings, Gower’s distance method is adequate when ordinal variables are included in the clustering. The chi-square test and analysis of variance (F-test) were used to determine the differences in baseline measurements according to 5 vaccination-related clusters. Continuous variables are shown as mean and standard deviation, whereas binary/categorical variables are shown as frequencies and column percentages. We conducted logistic regression to assess the association between mental health status and 5 clusters related to COVID-19 vaccination, and the adjusted odds ratio (ORs) and 95% confidence intervals (CIs) were reported. The ORs were adjusted for age, sex, marital status, education level, income level, current smoking/drinking status, regular physical activity, previous chronic disease history, and baseline study year (2013-2018). All analyses were conducted in R version 3.6.3 (R Core Team, Vienna, Austria)with the “cluster” and “fpc” packages.

## RESULTS

The participants were divided into 5 clusters for the cluster analysis. Table 1 shows the results of k-means clustering for the COVID-19 vaccine trust and intention questions. The response rates of each scale among the COVID-19 vaccine-related questions are shown in Supplementary Material 3 and 4 (mode value: trust [3] and “wait until it has been available for a while to see how it is working for other people,” 23.1% among the total). Approximately half the participants were classified into the moderate trust and high intention cluster (second cluster: n= 838, 47.4%). This was followed by the low trust and high intention cluster (first cluster: n= 439, 24.8%), high trust and high intention cluster (third cluster: n= 298, 16.9%), moderate trust and low intention cluster (fifth cluster: n = 99, 5.6%), and low trust and low intention cluster (fourth cluster: n= 94, 5.3%).

Table 2 shows the baseline characteristics of the participants for each of the 5 clusters. The majority of participants were female (65.3%), married and living with their spouse (87.2%), educated above college level (55.9%), current drinkers (74.0%), lacked regular physical activity (59.1%), and had no chronic disease history at baseline (56.8%). In the distribution by 5 clusters divided by the k-means method, the high trust and intention cluster (n= 298), especially, showed different characteristics compared with other clusters. Participants in the third cluster tended to be older (mean age, 53.65± 7.74 years) and had higher proportions of several categories: they were more likely to be male (53.6%), married (89.3%), high-income (household income, the fourth [richest] quartile= 35.2%), and current drinkers (78.2%), to engage in regular physical activities (49.3%), to have a chronic disease history (50.0%) (including hypertension and diabetes), and to show higher cognitive function, as assessed by the MMSE-DS (mean MMSE score, 28.01± 1.53). By contrast, the low trust and low intention cluster group (n= 94) tended to be younger (mean age, 47.65± 9.34 years), had a higher proportion of longer educational years (college or above, 68.1%), had a lower income level (household income, first quartile [lowest], 30.9%), had no chronic disease history (61.7%), and had high BDI-II scores (mean BDI score, 10.95± 9.11) compared to other clusters.

Table 3 shows the associations between mental health status (sleep quality, depression, general anxiety, post-traumatic stress, loneliness, and psychological resilience) and the COVID-19 vaccination clusters. Overall, mental health indices were significantly associated with the clusters after full adjustment (global p< 0.05), except the PSQI score (global p-value for the continuous PSQI score = 0.109; for PSQI categories, p = 0.068). Individuals with worse scores in the PHQ-9, GAD-7, ULS-6, CD-RISC-10 tended to show lower odds of being included in the second cluster (moderate trust and high intention) and third cluster (high trust and high intention) than in the reference group (first cluster: low trust and high intention). Regarding intention to receive COVID-19 vaccination, individuals with better mental health scores in the PSQI, PCL-5, ULS-6, and CD-RISC-10 tended to show lower odds of being included in the fourth cluster (low trust and high intention) than in the reference group.

As an additional analysis, we conducted a mediation analysis to explore indirect associations of each mental health index with the intention to receive COVID-19 vaccination through trust toward the vaccine. Although there was no statistically significant direct effect, worse mental health scores were directly associated with higher intention to receive COVID-19 vaccination, except for the PCL-5. In contrast, higher PHQ-9 and UCL-6 scores and lower CD-RISC scores were indirectly associated with lower intention (p< 0.05) (Supplementary Material 5).

## DISCUSSION

In this community-based longitudinal study, more than 10% of participants clustered into the low COVID-19 vaccine intention group (refusal or hesitant attitude toward vaccination), and approximately about 30% of the participants clustered into the group with low trust toward vaccine efficacy. We explored the pre-pandemic characteristics related to trust in and intention to receive vaccination. In addition, we assessed the associations of trust in and intention to receive vaccination with concurrent mental health status. Younger age, female, low-income level, irregular physical activity, and previous hypertension history were significantly related to negative trust or intention to receive COVID-19 vaccination. In a pandemic situation, middle-aged female with low income and sedentary behavior may be more hesitant to get vaccinated. The cluster of people with high trust and high intention to receive vaccination showed better mental health status compared to the low trust but high intention group.

According to previous findings, a majority of studies suggested that hesitancy or refusal of COVID-19 vaccination was related to specific socio-demographic characteristics. In a Unite States study, conducted in May 2020, individuals who reported refusal attitudes tended to exhibit specific characteristics, such as being African-American, younger, female, and having a low income level [10]. Another United States study investigated the acceptability of a COVID-19 vaccine (n= 2,006) where approximately 69% of participants reported their willingness to receive the vaccine. Individuals who were female, younger, and African-American tended to show low intention (willingness). By contrast, individuals who were married and living with their spouse, had high income, moderate or liberal political leanings, urban residence, and had underlying medical conditions, tended to show high intention [35]. Additionally, in this study, younger female with low socioeconomic status showed low trust and low intention compared to older male. Regarding physical or mental comorbidities, only the proportion of participants with a hypertension history at baseline differed significantly among the COVID-19 vaccine clusters (high trust and high intention group: 29.19%; low trust, and low intention group: 12.77%).

In this study, each mental health index was significantly associated with the clusters related to COVID-19 vaccination, except PSQI. Individuals with worse mental health status tend to show a higher proportion in the first cluster (low trust and high intention) than in the clusters. It is difficult to explain why mental health was associated with attitudes toward COVID-19 vaccination, but worry/fear related to the COVID-19 pandemic has been associated with depressive mood, anxiety, and lower resilience [36]; this relationship may manifest as acceptance and intention to receive vaccination [37,38]. We also observed a consistent pattern between worse mental health status and trust/intention toward the COVID-19 vaccine. Among those who intended to receive COVID-19 vaccination, individuals with better mental health showed higher trust (PHQ-9 score: second cluster, OR, 0.961; third cluster, OR, 0.942). However, among those with low trust toward the vaccine, individuals with better mental health showed lower intention (PHQ-9 score: fourth cluster, OR, 0.928). These results support the research hypothesis that trust and intention toward the vaccine should be considered simultaneously.

### Strengths and limitations

The strengths of our study include its various dimensions of mental health status, with scales measuring depressive symptoms, general anxiety symptoms, post-traumatic stress, loneliness, and resilience. This allowed us to estimate how trust in the vaccine and intention to receive vaccination correlated with each aspect of mental health status during the pandemic. While previous studies only explored factors influencing vaccination, our study is novel in that it focused on the association between trust in and intention to receive COVID-19 vaccination and current mental health.

Nevertheless, the study had some limitations that need to be considered when interpreting our results. First, information during COVID-19 was collected through a mobile/online survey. Although we used a mental health index that is valid in our population and similar populations with similar cultural backgrounds, there could be some measurement bias because online surveys generally have lower internal reliability or more insincere responses [39]. However, according to a previous systematic review (n= 33), the digital versions of self-report symptom scales showed high inter-format reliability and were comparable with pen and paper versions [40]. Second, the problem of selection bias and generalizability can be raised because there is a probability of higher participation of people more familiar with online surveys conducted on mobile phones; those unfamiliar with the digital environment may decline the initial assessment. The characteristics of included and excluded participants are shown in Supplementary Material 2. Third, according to previous vaccine hesitancy studies, individuals’ political orientation or ethnicity can affect vaccine hesitancy or refusal; such social characteristics, however, were not investigated in this study, remaining residual confounders. Fourth, the baseline study was conducted from 2013 to 2018, and the third mobile survey was carried out 3 years after the baseline. There could be some change during this gap period between the baseline and mobile survey. Finally, the study has limitations in temporal interpretation because the mobile survey (February 19 to March 12, 2021) was conducted before vaccination in the general population, which started on February 26, 2021. After the survey period, attitudes, intention, and trust regarding COVID-19 vaccination may be affected by the adverse events of both mRNA and viral vector COVID-19 vaccines. As representative examples, thrombosis with thrombocytopenia syndrome events associated with a viral vector vaccine were reported in March 2021. Subsequently, myocarditis and pericarditis events after mRNA COVID-19 vaccination were reported in various countries (April 2021) and many institutions, including the World Health Organization, European Medicines Agency, and Advisory Committee on Immunization Practices conducted causality assessments (May 2021). Furthermore, in Korea, cases of death after adverse events increased steadily (June: 98; July: 160; August: 213; September: 260). Many medical professionals have emphasized that the benefits outweigh the risks of vaccines, but public anxiety and concern about vaccines have increased more than before the reports of adverse events were issued.

Despite these limitations, our study has novelty in investigating the association between mental health and attitudes toward COVID-19 vaccination in a targeted population. By clustering participants with trust in and intention to receive the vaccination, we could obtain specific information on attitudes toward COVID-19 vaccination in association with socio-demographic characteristics and mental health status (during the pandemic). Furthermore, our findings support previous results that individuals with negative mental health status during the pandemic tend to have higher intention to receive COVID-19 vaccination, because a negative health status implies a higher level of stressors during COVID-19.

## Figures and Tables

**Table 1.** Definitions and proportions of each cluster

Group^1^	n (%)	COVID-19 vaccination	
		Trust-related question (included response)^2^	Intention-related question (included response)^3^
Cluster 1 (low trust and high intention)	439 (24.8)	Low reliability (0, 1, 2)	Yes (1, 2, 3)
Cluster 2 (moderate trust and high intention)	838 (47.4)	Moderate reliability (3, 4)	Yes (1, 2, 3)
Cluster 3 (high trust and high intention)	298 (16.9)	High reliability (4, 5)	Yes (1, 2, 3)
Cluster 4 (low trust and low intention)	94 (5.3)	Low reliability (0, 1)	No or Don’t know (3 ,4, 5)
Cluster 5 (moderate trust and low intention)	99 (5.6)	Moderate to high reliability (2, 3, 4, 5)	No or Don’t know (4, 5)

COVID-19, coronavirus disease 2019.

1 Five-clusters derived from k-means clustering (Gower’s distance and Forgy algorithm).

2 “How much do you trust the effects of the COVID-19 vaccine?”: (0)–strongly unreliable, (1), (2), (3), (4), (5)– strongly reliable.

3 “When a vaccine for COVID-19 is approved and widely available to anyone who wants it, would you like to get the vaccine?”: (1) “get the vaccine as soon as you can”; (2) “wait until it has been available for a while to see how it is working for other people”; (3) “only get the vaccine if you are required to do so for work, school, or other activities”; (4) “refuse”; and (5) “don’t know.”

**Table 2.** Baseline characteristics according to COVID-19 vaccination cluster

Characteristics		Total	Cluster^1^					
			1 (n=439)	2 (n=838)	3 (n=298)	4 (n=94)	5 (n=99)	p-value
Age at baseline (yr)		50.84±9.49	49.58±10.06	50.72±9.53	53.65±7.74	47.65±9.34	52.09±9.37	0.006
Sex								
	Male	613 (34.7)	130 (29.6)	285 (34.0)	159 (53.4)	23 (24.5)	16 (16.2)	<0.001
	Female	1,155 (65.3)	309 (70.4)	553 (66.0)	139 (46.6)	71 (75.5)	83 (83.8)	
Marital status								
	Married-living together	1,541 (87.2)	376 (85.7)	738 (88.1)	266 (89.3)	77 (81.9)	84 (84.9)	0.395
Education level								
	Elementary school	45 (2.6)	9 (2.1)	22 (2.6)	9 (3.0)	0 (0.0)	5 (5.1)	0.070
	Middle school	101 (5.7)	21 (4.8)	43 (5.1)	25 (8.4)	4 (4.3)	8 (8.1)	
	High school	633 (35.8)	171 (39.0)	299 (35.7)	98 (32.9)	26 (27.7)	39 (39.4)	
	College+	989 (55.9)	238 (54.2)	474 (56.6)	166 (55.7)	64 (68.1)	47 (47.5)	
Household income level								
	Q1	379 (21.4)	102 (23.2)	168 (20.1)	54 (18.1)	29 (30.9)	26 (26.3)	0.005
	Q2	588 (33.3)	151 (34.4)	293 (35.0)	80 (26.9)	29 (30.9)	35 (35.4)	
	Q3	305 (17.3)	81 (18.5)	138 (16.5)	59 (19.8)	9 (9.6)	18 (18.2)	
	Q4	496 (28.1)	105 (23.9)	239 (28.5)	105 (35.2)	27 (28.7)	20 (20.2)	
BMI (kg/m^2^)		23.72±3.00	23.71±3.11	23.63±2.97	23.88±2.67	23.66±3.50	24.07±3.16	0.264
Waist circumference (cm)		80.60±9.21	80.25±9.30	80.43±9.05	82.08±9.08	79.64±10.31	80.12±9.15	0.536
Current smoking status								
	No	1,576 (89.1)	397 (90.4)	739 (88.2)	259 (86.9)	86 (91.5)	95 (96.0)	0.079
	Yes	192 (10.9)	99 (11.8)	39 (11.8)	39 (13.1)	8 (8.5)	4 (4.0)	
Current drinking status								
	Former/non-drinkers	459 (26.0)	117 (26.6)	216 (25.8)	65 (21.8)	29 (30.9)	32 (32.3)	0.196
	Current drinkers	1,309 (74.0)	322 (73.4)	622 (74.2)	233 (78.2)	65 (69.2)	67 (67.7)	
Regular physical activities								
	No	1,044 (59.1)	265 (60.4)	513 (61.2)	151 (50.7)	52 (55.3)	63 (63.6)	0.017
	Yes	724 (40.9)	174 (39.6)	325 (38.8)	147 (49.3)	42 (44.7)	36 (36.4)	
Chronic disease history								
	No	1,005 (56.8)	256 (58.3)	486 (58.0)	149 (50.0)	58 (61.7)	56 (56.6)	0.115
	Yes	763 (43.2)	183 (41.7)	352 (42.0)	149 (50.0)	36 (38.3)	43 (43.4)	
Ever had hypertension								
	No	1,365 (77.2)	353 (80.4)	647 (77.2)	211 (70.8)	82 (87.3)	72 (72.7)	0.003
	Yes	403 (22.8)	86 (19.6)	191 (22.8)	87 (29.2)	12 (12.7)	27 (27.3)	
SBP (mmHg)		117.63±14.89	117.70±15.54	116.98±14.59	119.87±14.16	114.82±14.64	118.79±16.18	0.469
DBP (mmHg)		75.56±9.75	75.58±9.77	75.23±9.84	76.73±9.38	74.05±9.62	76.22±9.90	0.522
Ever had diabetes								
	No	1,639 (92.7)	413 (94.1)	776 (92.6)	269 (90.3)	88 (93.6)	93 (93.9)	0.381
	Yes	129 (7.3)	26 (5.9)	62 (7.4)	29 (9.7)	6 (6.4)	6 (6.1)	
Fasting glucose (mg/dL)		90.20±17.27	91.57±19.69	92.94±15.86	89.63±15.12	92.29±19.14	91.40±18.25	0.247
Total cholesterol (mg/dL)		198.42±35.59	197.61±36.16	199.76±35.60	194.95±34.49	201.71±36.93	197.96±34.69	0.878
Triglyceride (mg/dL)		124.47±84.22	119.42±74.23	125.17±84.20	135.80±107.60	113.00±63.75	117.65±55.56	0.772
HDL cholesterol (mg/dL)		58.04±14.65	58.15±14.01	58.42±15.05	56.52±14.10	59.22±14.04	57.80±16.02	0.580
hsCRP (mg/dL)		1.45±4.15	1.65±5.08	1.31±3.31	1.28±2.47	2.53±8.61	1.29±3.34	0.983
Social network characteristics								
	Size	4.07±1.6	4.04±1.55	4.1±1.64	4.01±1.56	4.08±1.58	4.18±1.61	0.687
	Intimacy (mean)	3.21 ±0.58	3.17±0.6	3.22±0.57	3.19±0.59	3.41±0.48	3.18±0.55	0.107
	Female proportion	0.59±0.27	0.61±0.26	0.6±0.27	0.53±0.27	0.61±0.26	0.63±0.25	0.201
	Kin proportion	0.51±0.3	0.52±0.31	0.52±0.3	0.47±0.3	0.51±0.31	0.5±0.32	0.168
	Health-communication-level (mean)	1.33±0.38	1.37±0.42	1.32±0.37	1.3±0.35	1.28±0.38	1.36±0.41	0.091
BDI-II score (range: 0-63)^2^		10.26±6.90	9.10±6.63	8.21±6.35	9.06±7.19	10.95±9.11	9.34±6.88	0.251
MMSE-DS score (range: 0-30)^2^		27.84±1.68	27.63±1.77	27.87±1.71	28.01±1.53	28.00±1.51	27.73±1.63	0.138

Values are presented as mean±standard deviation or number (%). COVID-19, coronavirus disease 2019; BMI, body mass index; SBP, systolic blood pressure; DBP, diastolic blood pressure; HDL, high-density lipoprotein; hsCRP, high-sensitivity C-reactive protein; BDI, Beck Depression Inventory; MMSE-DS, Mini-Mental State Examination for Dementia Screening.

1 Participants divided into five-clusters due to k-means clustering with the Forgy algorithm.

2 Number of participants: BDI-II, 1,767 participants; MMSE-DS, 1,112 participants.

**Table 3.** Association between mental health and COVID-19-related clusters

Mental health		COVID-19-related cluster^1^					Global p-value
		1 (n=439)	2 (n=838)	3 (n=298)	4 (n=94)	5 (n=99)	
PSQI							
	Continuous	1.00 (reference)	0.97 (0.94, 1.01)	0.94 (0.90, 0.99)	0.93 (0.86, 1.00)	0.98 (0.92, 1.04)	0.109
	Categorical (cut-off: 8.5)	1.00 (reference)	0.68 (0.50, 0.91)	0.64 (0.42, 0.96)	0.69 (0.38, 1.28)	0.95 (0.56, 1.61)	0.068
PHQ-9							
	Continuous	1.00 (reference)	0.96 (0.94, 0.98)	0.94 (0.91, 0.97)	0.97 (0.93, 1.01)	0.96 (0.92, 1.00)	0.001
	Categorical (cut-off: 15)	1.00 (reference)	0.65 (0.48, 0.87)	0.62 (0.41, 0.95)	0.88 (0.50, 1.55)	0.70 (0.39, 1.23)	0.043
GAD-7							
	Continuous	1.00 (reference)	0.97 (0.94, 0.99)	0.94 (0.90, 0.98)	0.96 (0.90, 1.02)	0.96 (0.91, 1.02)	0.027
	Categorical (cut-off: 15)	1.00 (reference)	0.62 (0.41, 0.94)	0.52 (0.28, 0.97)	0.51 (0.19, 1.33)	0.79 (0.37, 1.71)	0.119
PCL-5							
	Continuous	1.00 (reference)	0.99 (0.98, 1.00)	0.98 (0.97, 0.99)	0.97 (0.95, 0.99)	1.00 (0.98, 1.01)	0.009
	Categorical (cut-off: 38)	1.00 (reference)	0.66 (0.37, 1.16)	0.58 (0.25, 1.34)	0.40 (0.09, 1.76)	1.37 (0.56, 3.36)	0.237
ULS-6							
	Continuous	1.00 (reference)	0.94 (0.91, 0.97)	0.94 (0.90, 0.98)	0.93 (0.86, 0.99)	0.99 (0.93, 1.05)	0.003
CD-RISC-10							
	Continuous	1.00 (reference)	1.01 (1.00, 1.03)	1.04 (1.02, 1.06)	1.04 (1.01, 1.07)	1.02 (0.99, 1.05)	0.001

Values are presented as odds ratio (95% confidence interval). COVID-19, coronavirus disease 2019; PSQI, Pittsburgh Sleep Quality Index; PHQ, Patient Health Questionnaire; GAD, Generalized Anxiety Disorder; PCL, Post-traumatic Stress Disorder Checklist for the Diagnostic and Statistical Manual Disorders; ULS, UCLA Loneliness Scale; CD-RISC, Connor-Davidson Resilience Scale.

1 Adjusted by age, sex, years at enrollment, marital status, education level, income level (quartile), current smoking/drinking status, regular physical activity (yes/no), previous chronic disease history (yes/no), previous hypertension, and diabetes history.

## SUPPLEMENTARY MATERIALS

Supplementary Material 1. Collected information in COVID-19 mobile survey

Supplementary Material 2 Characteristics between included and excluded participants

Supplementary Material 3 Response rate of trust and intention to COVID-19 vaccine.

Supplementary Material 4 Mosaic plot of trust and intention to COVID-19 vaccine.

Supplementary Material 5 Results from mediation analysis (X: mental health status, M: Trust to the COVID-19 vaccine, Y: Intention to the vaccine)
