# Supplementary material for: Physical and mental health characteristics related to trust in and intention to receive COVID-19 vaccination: results from a Korean community-based longitudinal study
Source: Epidemiol Health. 2022 Aug 3;44:e2022064. doi: 10.4178/epih.e2022064 (PMC9943634; doi:10.4178/epih.e2022064)
Supplement: Supplementary Material 1. — Collected information in COVID-19 mobile survey [file epih-44-e2022064-suppl1.docx]

**Supplementary Material 1.** Collected information in COVID-19 mobile survey

|  | **1st mobile survey**  **(N=1,970)** |  | **2nd mobile survey**  **(N=1,905)** |  | **3rd mobile survey**  **(N=)** |
| --- | --- | --- | --- | --- | --- |
| **Category** | (March, 2020) |  | (August, 2020) |  | (Feb-March, 2021) |
| **Basic information** | Disease history |  | Disease history |  | Disease history |
| **Mental health status during**  **pandemic** | Stress |  | Stress |  | Stress |
|  | PSQI* |  | PSQI |  | PSQI |
|  | GAD-7 |  | GAD-7 |  | GAD-7 |
|  | PHQ-9 |  | PHQ-9 |  | PHQ-9 |
|  | PCL-5 |  | PCL-5 |  | PCL-5 |
|  | UCL-6 |  | UCL-6 |  | UCL-6 |
|  | CD-RISC-10 |  | CD-RISC-10 |  | CD-RISC-10 |
|  | Suicide plan/attemp |  | Suicide plan/attemp |  | Suicide plan/attemp |
| **COVID-19 related questionnaire** | Cognition/Prevention |  | Cognition/Prevention |  | Cognition/Prevention |
|  | Support/Information |  | Support/Information |  | Support/Information |
|  |  |  |  |  | Vaccine |
|  |  |  |  |  | Social distance |
