# Supplementary material for: Physical and mental health characteristics related to trust in and intention to receive COVID-19 vaccination: results from a Korean community-based longitudinal study
Source: Epidemiol Health. 2022 Aug 3;44:e2022064. doi: 10.4178/epih.e2022064 (PMC9943634; doi:10.4178/epih.e2022064)
Supplement: Supplementary Material 2 — Characteristics between included and excluded participants [file epih-44-e2022064-suppl2.docx]

**Supplementary Material 2.** Characteristics between included and excluded participants

| **Variable** | **Excluded** | **Included** |  |
| --- | --- | --- | --- |
|  | **(N=2,292)** | **(N=1,768)** | **p-value** |
| Age at baseline, years | 51.67±9.23 | 50.84 ± 9.49 | 0.0052 |
| Gender |  |  |  |
| Male | 813(35.5%) | 613(34.67%) | 0.5968 |
| Female | 1479(64.5%) | 1,155(65.33%) |  |
| Marital status |  |  |  |
| Married-living together | 1938(84.6%) | 1541(87.16%) | 0.0066 |
| Education level |  |  |  |
| Elementary school | 185(8.07%) | 45(2.55%) | <.001 |
| Middle school | 249(10.86%) | 101(5.71%) |  |
| High school | 952(41.54%) | 633(35.80%) |  |
| College + | 906(39.53%) | 989(55.94%) |  |
| Household income level |  |  |  |
| Q1 | 646(28.2%) | 379(21.44%) | <.001 |
| Q2 | 757(33.0%) | 588(33.26%) |  |
| Q3 | 363(15.8%) | 305(17.25%) |  |
| Q4 | 526(23.0%) | 496(28.05%) |  |
| BMI, kg/m^2^ | 24.02±3.10 | 23.72 ± 3.00 | 0.0021 |
| Waist circumference, cm | 81.41±9.03 | 80.60 ± 9.21 | 0.005 |
| Current smoking status |  |  |  |
| No | 1,928(84.1%) | 1,576(89.14%) | <.001 |
| Yes | 364(15.9%) | 192(10.86%) |  |
| Current drinking status |  |  |  |
| Former/Non-drinkers | 631(27.5%) | 459(25.96%) | 0.2523 |
| Current drinkers | 1,661(72.5%) | 1,309(74.04%) |  |
| Regular physical activities |  |  |  |
| No | 1,366(59.6%) | 1,044(59.05%) | 0.7241 |
| Yes | 926(40.4%) | 724(40.95%) |  |
| Chronic disease history |  |  |  |
| No | 1,268(55.3%) | 1,005(56.84%) | 0.333 |
| Yes | 1,024(44.7%) | 763(43.16%) |  |
| Hypertension history |  |  |  |
| No | 1,629(71.1%) | 1,365(77.2%) | <.001 |
| Yes (Diagnosis, medication, or 140/90mmHg) | 663(28.9%) | 403(22.8%) |  |
| Diabetes history |  |  |  |
| No | 2,068(90.2%) | 1,620(91.6%) | 0.1247 |
| Yes (Diagnosis, medication, or 126mg/dL) | 224(9.8%) | 148(8.4%) |  |
| Total cholesterol, mg/dL | 198.50±35.19 | 198.42 ± 35.59 | 0.9741 |
| Triglyceride, mg/dL | 130.60±90.33 | 124.47 ± 84.22 | 0.0263 |
| HDL cholesterol, mg/dL | 57.22± 14.6895 | 58.04 ± 14.65 | 0.0792 |
| hsCRP, mg/dL | 1.43±3.639 | 1.45 ± 4.15 | 0.9213 |
| Social network characteristics |  |  |  |
| Size | 3.916±1.568 | 4.07 ± 1.6 | 0.0078 |
| Intimacy (mean) | 3.152±0.595 | 3.21 ± 0.58 | 0.0032 |
| Female proportion | 0.587±0.274 | 0.59 ± 0.27 | 0.597 |
| Kin proportion | 0.508±0.309 | 0.51 ± 0.3 | 0.8909 |
| Health communication level (mean) | 1.35±0.390 | 1.33 ± 0.38 | 0.1075 |
| BDI-II score (range: 0-63) | 10.26±7.60 | 9.34 ± 6.88 | <.001 |
| MMSE-DS score (range: 0-30) | 27.30±1.95 | 27.84 ± 1.68 | <.001 |
| *Social network characteristics; 2,280; 1,756 participants* | |  |  |
| *BDI-II; 2,291; 1,767 participants* |  |  |  |
| *MMSE-DS; 1,545; 1,112 participants* |  |  |  |
