# Supplementary material for: Physical and mental health characteristics related to trust in and intention to receive COVID-19 vaccination: results from a Korean community-based longitudinal study
Source: Epidemiol Health. 2022 Aug 3;44:e2022064. doi: 10.4178/epih.e2022064 (PMC9943634; doi:10.4178/epih.e2022064)
Supplement: Supplementary Material 3 — Response rate of trust and intention to COVID-19 vaccine. [file epih-44-e2022064-suppl3.docx]

**Supplementary Material 3.** Response rate of trust and intention to COVID-19 vaccine.

|  | **Intention related question**, Frequency(%) | | | | | |
| --- | --- | --- | --- | --- | --- | --- |
| **Trust related question** | Get the vaccine as soon as you can | Wait until it has been available for a while to see how it is working for other people | Only get the vaccine if you are required to do so for work | Refused | Don't know | **Total** |
| Trust (0 - strongly untrust) | 2 (0.1%) | 28 (1.6%) | 6 (0.3%) | 24 (1.4%) | 5 (0.3%) | 65 |
| Trust (1) | 9 (0.5%) | 91 (5.2%) | 21 (1.2%) | 19 (1.1%) | 18 (1%) | 158 |
| Trust (2) | 34 (1.9%) | 230 (13.1%) | 42 (2.4%) | 9 (0.5%) | 35 (2%) | 350 |
| Trust (3) | 195 (11.1%) | 406 (23.1%) | 82 (4.7%) | 11 (0.6%) | 34 (1.9%) | 728 |
| Trust (4) | 206 (11.7%) | 131 (7.5%) | 19 (1.1%) | 2 (0.1%) | 4 (0.2%) | 362 |
| Trust (5 - strongly trust) | 73 (4.2%) | 15 (0.9%) | 1 (0.1%) | 0 (0%) | 4 (0.2%) | 93 |
| **Total** | 519 | 901 | 171 | 65 | 100 | **1,756** |
