# Supplementary figures and images for: Physical and mental health characteristics related to trust in and intention to receive COVID-19 vaccination: results from a Korean community-based longitudinal study
Source: Epidemiol Health. 2022 Aug 3;44:e2022064. doi: 10.4178/epih.e2022064 (PMC9943634; doi:10.4178/epih.e2022064)

**Supplementary Material 4.** Mosaic plot of trust and intention to COVID-19 vaccine.

***
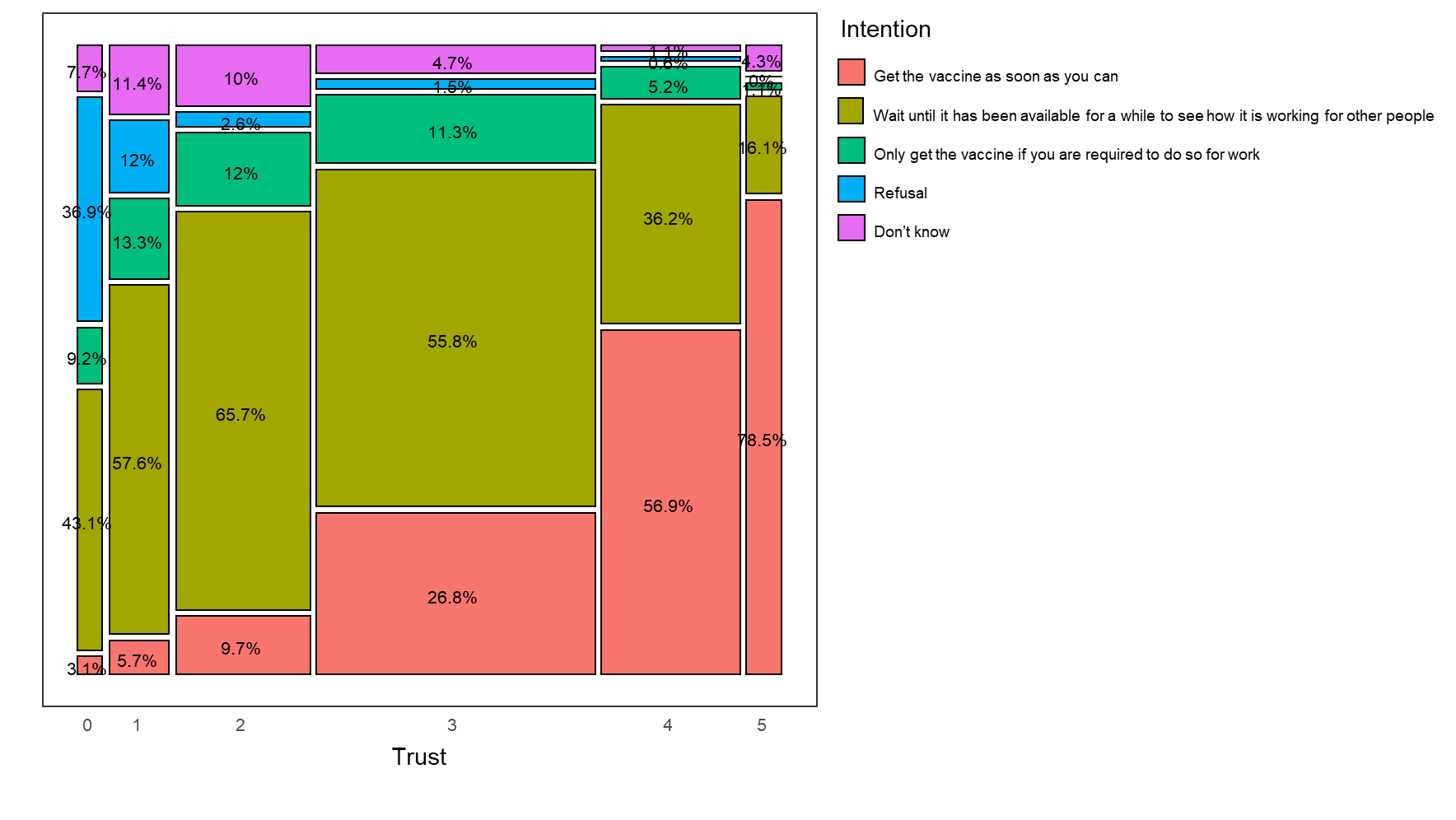
***

Supplement: Supplementary Material 4 — Mosaic plot of trust and intention to COVID-19 vaccine. [file epih-44-e2022064-suppl4.docx]
