# Supplementary material for: Physical and mental health characteristics related to trust in and intention to receive COVID-19 vaccination: results from a Korean community-based longitudinal study
Source: Epidemiol Health. 2022 Aug 3;44:e2022064. doi: 10.4178/epih.e2022064 (PMC9943634; doi:10.4178/epih.e2022064)
Supplement: Supplementary Material 5 — Results from mediation analysis (X: mental health status, M: Trust to the COVID-19 vaccine, Y: Intention to the vaccine) [file epih-44-e2022064-suppl5.docx]

**Supplementary Material 5.** Results from mediation analysis (X: mental health status, M: Trust to the COVID-19 vaccine, Y: Intention to the vaccine)

| **Mental health** (X) | **Mediation analysis** (M: Trust, Y: Intention to COVID-19 vaccine) | | | | | | | |
| --- | --- | --- | --- | --- | --- | --- | --- | --- |
|  | Total effect | |  | Direct effect  (X>Y) | |  | Indirect effect (X>M>Y) | |
|  | Beta (SE) | p-value |  | Beta (SE) | p-value |  | Beta (SE) | p-value |
| **PSQI** | 0.003 (0.02) | 0.894 |  | 0.02 (0.02) | 0.405 |  | -0.01 (0.01) | 0.149 |
| **PHQ-9** | -0.01 (0.02) | 0.734 |  | 0.02 (0.02) | 0.275 |  | -0.03 (0.01) | 0.004 |
| **GAD-7** | 0.001 (0.02) | 0.983 |  | 0.02 (0.02) | 0.346 |  | -0.02 (0.01) | 0.051 |
| **PCL-5** | -0.01 (0.02) | 0.600 |  | -0.002 (0.02) | 0.942 |  | -0.01 (0.01) | 0.278 |
| **UCL-6** | -0.02 (0.02) | 0.457 |  | 0.005 (0.02) | 0.823 |  | -0.02 (0.01) | 0.034 |
| **CD-RISC-10** | -0.01 (0.02) | 0.820 |  | -0.03 (0.02) | 0.1207 |  | 0.03 (0.01) | 0.011 |

***(Note)*** adjusted for age, sex, study year, education, income, marital status, regular physical activity, current smoking and drinking status, hypertension history, diabetes history.
